# Post-Traumatic Stress Disorder (PTSD) Is Associated with Increased Physical Skin Symptom Burden Following Severe Burn Injuries: Subgroup Analysis of a Multicenter Prospective Cohort

**DOI:** 10.3390/ebj6030043

**Published:** 2025-08-08

**Authors:** Felix J. Klimitz, Martin Aman, Hubert Neubauer, Annette Stolle, Hans Ziegenthaler, Tobias Niederegger, Adriana C. Panayi, Gabriel Hundeshagen, Ulrich Kneser, Leila Harhaus

**Affiliations:** 1 Department of Surgery, Division of Plastic Surgery, Yale School of Medicine, New Haven, CT 06510, USA; 2 Department of Hand-, Plastic and Reconstructive Surgery, Microsurgery, Burn Center, BG Trauma Center Ludwigshafen, University of Heidelberg, 67071 Ludwigshafen, Germany; 3 Department of Hand, Replantation, and Microsurgery, BG Trauma Center Berlin, Chair of Hand, Replantation, and Microsurgery, Charité—Universitätsmedizin Berlin, 10117 Berlin, Germany; 4 Rehabilitation Center, Graefliche Kliniken-Moritz Klinik GmbH, 07639 Bad Klosterlausnitz, Germany; 5 Department of Oral and Maxillofacial Surgery, Charité—Universitätsmedizin Berlin, Corporate Member of Freie Universität Berlin, Humboldt-Universität zu Berlin, and Berlin Institute of Health, 10099 Berlin, Germany

**Keywords:** burn injuries, post-traumatic stress disorder (PTSD), physical symptoms, psychosomatic interactions, rehabilitation, quality of life

## Abstract

Background: Severe burn injuries often lead to lasting physical and psychological consequences. Post-traumatic stress disorder (PTSD) is common among burn survivors and may be influenced by persistent somatic complaints. This study examined whether PTSD is associated with a higher burden of physical symptoms during and after inpatient rehabilitation. Methods: We conducted a subgroup analysis of a multicenter prospective cohort study involving 103 adult burn patients in inpatient rehabilitation. Based on Impact of Event Scale—Revised (IES-R) scores and clinical evaluation, patients were grouped as PTSD (n = 43) or No PTSD (n = 60). Physical symptoms assessed included skin dryness (xerosis), temperature sensitivity (cold/heat), numbness, skin tightness, and increased sweating. Results: Patients with PTSD reported significantly more physical symptoms at follow-up than those without PTSD: xerosis (74% vs. 50%, *p* = 0.03), cold sensitivity (61% vs. 35%, *p* = 0.02), heat sensitivity (63% vs. 39%, *p* = 0.03), numbness (63% vs. 33%, *p* = 0.006), skin tightness (82% vs. 52%, *p* = 0.004), and sweating (45% vs. 19%, *p* = 0.01). PTSD patients also had more severe burns, reflected in higher full-thickness TBSA (2% vs. 0%, *p* = 0.03) and elevated ABSI scores (median 6 vs. 5, *p* = 0.04). Conclusion: PTSD is associated with a higher and more persistent burden of physical skin symptoms after severe burns. These findings underscore the importance of early PTSD screening and integrated psychological-somatic rehabilitation to improve long-term recovery and quality of life.

## 1. Introduction

Severe burn injuries are often associated with complex long-term sequelae, making inpatient rehabilitation essential for optimizing recovery [1]. The goals of burn rehabilitation are multifaceted ranging from the restoration of physical function (improving mobility, self-care independence, and activities of daily living) to addressing psychosocial needs [2,3]. Rehabilitation begins early—often alongside acute care—and continues through scar maturation to help patients regain independence and quality of life [4]. This involves not only physical reconditioning and management of pain and scarring but also psychological support to cope with trauma and adapt to life after injury [5,6,7].

Post-traumatic stress disorder (PTSD) is a frequent psychiatric diagnosis in burn survivors, reflecting the extreme trauma of the injury and treatment experience [8]. Meta-analyses estimate that roughly one in five burn survivors meets the diagnostic criteria for PTSD within the first couple of years after injury [9]. However, rates can vary depending on assessment and timing, with some studies reporting even higher early post-burn PTSD symptom prevalence. This high prevalence underscores the clinical significance of PTSD in the burn population. Importantly, the presence of PTSD may adversely impact rehabilitation outcomes: for example, approximately 45% of burn patients experience significant psychological distress in the first two years post-injury, and severe PTSD shortly after discharge is associated with poorer functional, psychological, and social recovery outcomes [10]. Moreover, it has been shown in patients with facial burns that even small and well-healing burn wounds can result in persistent psychological distress and reduced quality of life over the medium and long term [11]. PTSD in burn survivors is therefore not only common but also potentially detrimental to the overall rehabilitation trajectory and quality of life.

A growing body of evidence suggests that PTSD and related psychological factors may exacerbate the physical symptom burden following burns through psychosomatic interactions [10,12,13]. Adult burn survivors often endure persistent physical symptoms during inpatient rehabilitation, including skin-related issues such as chronically dry skin and abnormal sensitivity to temperature changes, as well as neuropathic and musculoskeletal complaints ranging from pruritus to joint and residual burn pain [14]. Many of these symptoms are direct consequences of the burn injury—for instance, burned skin loses sweat and oil glands, thus requiring regular moisturizing. Pruritus and pain are especially prevalent problems that can persist or even worsen during rehabilitation [15,16]. Mechanistically, PTSD may amplify these somatic symptoms via sustained stress responses and hyperarousal [6,15]. Chronic psychological stress is known to heighten the perception of pain and other sensations; indeed, anxiety and trauma-related disorders can lower the threshold for pain and itch, leading patients to experience these symptoms more intensely [17,18]. There is convincing evidence of an entangled, bidirectional relationship between chronic pain and PTSD in trauma survivors, and emerging data indicate that PTSD symptoms are also associated with worse post-burn pruritus severity [13,19,20]. In addition, individuals with PTSD may exhibit hypervigilance to bodily discomfort and engage in avoidant behaviors, such as reduced participation in therapy or movement due to fear of pain, which can further contribute to complications such as joint stiffness and pain [21,22]. Through these psychosomatic pathways, PTSD has the potential to increase the physical symptom burden during burn rehabilitation significantly.

The present study aimed to explore the association between clinically diagnosed PTSD and the burden of physical skin symptoms during inpatient rehabilitation after severe burn injury. Given the complex and potentially bidirectional relationship between PTSD and physical symptoms, we analyze this heterogeneous, multi-center sample to determine whether PTSD contributes to greater physical symptom burden in the rehabilitation setting, thereby providing insights that could inform comprehensive and multidisciplinary burn care and targeted interventions for this vulnerable subgroup.

## 2. Materials and Methods

### 2.1. Study Design

We performed subgroup analysis embedded within a previously published prospective multicenter, non-inferiority trial, which evaluated the effectiveness of a burn-specific rehabilitation program structured around the International Classification of Functioning, Disability, and Health (ICF) framework [5]. The original trial, conducted at two centers offering multidisciplinary inpatient care for burn injuries, systematically assessed physical and psychological outcomes over time [23]. Included patients had varying intervals between their burn injury and the start of rehabilitation, providing a diverse range of recovery timelines. The present analysis presents unpublished data, specifically focusing on differences in physical skin complaints between patients with a diagnosis of PTSD and those without.

### 2.2. Patient and Burn Characteristics

Demographic, clinical, and injury-related variables were assessed for all patients enrolled in the study. Demographic information included age, sex, and body mass index (BMI). Injury characteristics encompassed burn etiology (e.g., flame, scald, contact, chemical, electrical), total body surface area (TBSA) affected, and depth of injury categorized into superficial partial, deep partial, and full-thickness burns. The Abbreviated Burn Severity Index (ABSI) was calculated, providing a composite measure of burn severity based on age, sex, TBSA, presence of full-thickness burns, and inhalation injury. Additional clinical variables included the presence of inhalation injury, length of acute hospital stay, and duration of inpatient rehabilitation. These variables were extracted from standardized medical documentation completed at the time of admission to rehabilitation.

### 2.3. PTSD Classification

PTSD diagnosis in this study was established using the Impact of Event Scale-Revised (IES-R), a validated and widely used self-report instrument designed to assess post-traumatic stress symptoms [24]. The IES-R evaluates three core symptom domains of PTSD: intrusion, avoidance, and hyperarousal. A composite score was calculated by summing the responses across all items. A total IES-R score above the established cutoff of 33 was considered indicative of clinically significant PTSD symptoms, consistent with prior validation studies in burn populations [25,26]. Patients exceeding this threshold underwent further evaluation by an experienced psychologist to confirm the diagnosis of PTSD. The assessment was performed at the beginning of inpatient rehabilitation, which followed the acute hospital care stay (mean length 36.2 days). Based on this evaluation, patients were classified into two groups: those with a clinical diagnosis of PTSD and those without.

### 2.4. Physical Complaints Assessment

Physical skin symptoms were assessed at the beginning (T1) and end of burn-specific inpatient rehabilitation (T2), as well as 3 months (T3) and 12 months (T4) after discharge from rehabilitation, using a standardized checklist. The complaints analyzed in this study included skin dryness (xerosis) in the area of the burn or skin graft donor site, abnormal sensitivity to temperature (heat and cold), numbness, skin tightness, and increased propensity to sweat. Skin tightness describes the subjective experience of restricted or stiff skin. Sensitivity to heat or cold was defined as a heightened reaction to environmental temperature stimuli. Each of these symptoms was recorded in binary form (present or absent) and analyzed independently.

### 2.5. Statistical Analysis

All statistical analyses were conducted using R version 4.3.2 (R Foundation for Statistical Computing, Vienna, Austria). Descriptive statistics were used to summarize patient characteristics and symptom frequencies within each PTSD classification group. Categorical variables, including the presence of individual complaints, were expressed as counts and percentages. Continuous or ordinal data were summarized using medians and interquartile ranges. Continuous variables were assessed for normality using summary statistics and visual inspection; non-normally distributed variables were compared using the Mann–Whitney U test to evaluate differences in medians between groups. Categorical variables were analyzed using Fisher’s exact test due to small sample sizes or low expected cell counts. Welch’s two-sample *t*-test was applied to compare group means, which does not assume equal variances. A two-tailed *p*-value of less than 0.05 was considered statistically significant.

## 3. Results

### 3.1. Demographics and Injury Characteristics

A total of 103 patients undergoing inpatient burn rehabilitation were included, of whom 43 (41.7%) had a clinical diagnosis of PTSD. Patients with PTSD were slightly older than those without (median age 48, IQR 33–56 vs. 42 years, IQR 39–56), though this difference was not statistically significant (*p* = 0.39). The mean body mass index (BMI) was similar between groups (27.2 ± 4.7 kg/m^2^ vs. 27.1 ± 5.8 kg/m^2^; *p* = 0.91). While all patients without PTSD were male (n = 60), the PTSD group included four female patients (*p* = 0.03). Injury mechanism did not significantly differ between groups, with flame burns being the most common cause in both (28.3% vs. 32.6%; *p* = 0.67). Regarding TBSA, the prevalence of partial thickness burns did not differ between groups (8% vs. 6%, *p* = 0.31), while full thickness burns were higher in the PTSD group (2% vs. 0%, *p* = 0.03). A total of 26 patients in the PTSD group (60%) and 23 patients in the group without PTSD (38%) sustained full-thickness burns. Median Abbreviated Burn Severity Index (ABSI) scores were higher in the PTSD group (6 vs. 5, *p* = 0.04); however, this difference, while statistically significant, falls within the mild to moderate ABSI severity category and may not reflect a meaningful clinical distinction. There was no statistically significant difference regarding inhalation injury between the two groups (8.3% vs. 20.9%, *p* = 0.08). In addition, the median length of acute hospital stay and inpatient rehabilitation duration did not differ between the two groups (29 vs. 23 days, *p* = 0.16, and 5 vs. 3 weeks, *p* = 0.09, respectively). A detailed overview of patient and injury characteristics is provided in Table 1.

### 3.2. Physical Symptom Burden of the Entire Cohort

At the beginning of inpatient rehabilitation, patients exhibited a substantial burden of physical symptoms related to their burn injuries. The most frequently reported complaint was skin tightness, affecting 87% of patients, followed by xerosis (67%) and skin fragility (63%). Sensory disturbances were also common, with 44% of patients reporting sensitivity to heat, 40% reporting numbness, and 36% experiencing sensitivity to cold. A smaller proportion of patients (31%) noted an increased propensity to sweat. Patients continued to report a high burden of physical symptoms throughout the 12-month follow-up period. At T4, 60% of patients still experienced xerosis, 46% reported cold sensitivity, and 49% noted sensitivity to heat. Skin fragility remained prevalent in 64% of patients, while 46% continued to report numbness. Although skin tightness showed a gradual decrease from 87% at baseline to 64% at T4, it remained the most common complaint. Propensity to sweat was again the least frequently reported symptom, affecting 29% of patients at T4.

### 3.3. Impact of PTSD Status

**Figure 1:**
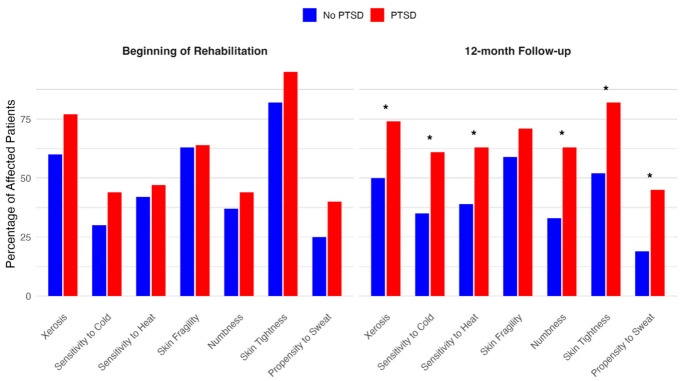
Comparison of physical symptom burden at the beginning of rehabilitation and 12-month follow-up between patients with and without PTSD. Asterisks (*) indicate statistically significant differences between groups (*p* < 0.05).

Across all symptom categories, patients with PTSD consistently had a higher symptom burden than those without PTSD, both at the onset of inpatient rehabilitation and at all follow-up time points. While overall symptom prevalence tended to decrease over time, significant residual burden remained at T4, particularly among PTSD patients. Figure 1 shows a comparison of physical symptom burden between T1 and T4 in both groups. While this pattern was observed for all assessed symptoms, statistically significant differences particularly emerged at the 12-month follow-up (T4) in several domains. Xerosis was reported by 74% of patients with PTSD versus 50% of those without (*p* = 0.03), and sensitivity to cold was present in 61% of patients with PTSD compared to 35% in those without (*p* = 0.02). Similarly, sensitivity to heat was more frequent among patients with PTSD (63% vs. 39%, *p* = 0.03). Notably, numbness and skin tightness also showed significant between-group differences at multiple timepoints: at T3, numbness was reported by 58% of PTSD patients versus 33% without (*p* = 0.01), and at T4, this difference persisted (63% vs. 33%, *p* = 0.006). Skin tightness at T3 and T4 was also more prevalent in the PTSD group (87% vs. 69%, *p* = 0.04; and 82% vs. 52%, *p* = 0.004, respectively). Additionally, a significantly greater proportion of patients with PTSD had an increased propensity to sweat at T4 (45% vs. 19%, *p* = 0.01). In patients without PTSD, most skin-related symptoms showed a general trend toward improvement over the 12-month follow-up period. Xerosis decreased slightly from 60% at the beginning of rehabilitation (T1) to 50% at 12 months (T4). Sensitivity to cold and heat remained relatively stable, with cold sensitivity ranging from 30% at T1 to 35% at T4, and heat sensitivity from 42% to 39%. Skin fragility remained consistent across all time points, with only a mild decrease from 63% (T1) to 59% (T4) over time. Notably, numbness and skin tightness showed a more marked reduction: numbness decreased from 37% at T1 to 33% at T4, and skin tightness declined from 82% at T1 to 52% at T4. Propensity to sweat also decreased from 25% at baseline to 19% at 12 months. These findings suggest that, in the absence of PTSD, physical skin symptoms tend to gradually improve during the course of recovery. An overview of the physical symptom burden over time, stratified by PTSD status, is presented in Table 2.

The above bar charts display the percentage of patients affected by each reported physical symptom at two timepoints: the beginning of inpatient rehabilitation and at 12-month follow-up. Percentages are based on available patient data at each time point (PTSD group: beginning of rehabilitation, n = 43; 12-month follow-up, n = 38. No PTSD group: beginning of rehabilitation, n = 60; 12-month follow-up, n = 54). An asterisk (*) indicates a statistically significant difference between the PTSD and No PTSD groups at *p* < 0.05.

## 4. Discussion

This study aimed to investigate whether PTSD is associated with an increased burden of physical symptoms following severe burn injuries. A key finding was that patients with PTSD consistently had higher rates of physical skin symptoms across all measured domains, including xerosis, temperature sensitivity, numbness, skin tightness, and increased propensity to sweat. Notably, this increased symptom burden persisted even one year after rehabilitation, underscoring the long-term impact PTSD may have on burn recovery.

Although the overall burn severity in our cohort was moderate (mean TBSA 15%, range 6–25%), our analysis revealed that patients with PTSD had relatively more severe injuries compared to those without PTSD, as indicated by a significantly higher proportion of full-thickness burns and elevated ABSI scores, both of which reflect more extensive and complex injuries. These findings are consistent with the hypothesis that more severe and disfiguring injuries may increase the risk of PTSD, either by intensifying the traumatic experience itself or by contributing to persistent somatic and functional impairments that serve as ongoing psychological stressors. Previous studies have confirmed that larger TBSA, deeper burns, and longer hospitalizations are associated with greater risk of depression, anxiety, PTSD, and impaired quality of life [6,8,27]. However, recent evidence also shows that even small burns can result in substantial psychological morbidity [11]. While the majority of the literature supports a positive association between burn severity and PTSD risk, it is noteworthy that some studies have found other factors, such as perceived life threat, pre-injury mental health status, and body image dissatisfaction, to be more predictive of PTSD than burn severity itself [10].

In addition, we found an increased physical symptom burden in patients with PTSD. These findings align well with prior research demonstrating heightened somatic symptoms in patients with PTSD. Multiple studies demonstrate that burn survivors with PTSD or significant posttraumatic stress symptoms experience higher rates and greater severity of somatic symptoms, including pain, pruritus, and neuropathic pain, compared to those without PTSD, with these symptoms often co-occurring and remaining elevated up to 18 months post-injury [12,13,15,27,28]. Recent large database analyses also confirm that PTSD in burn patients is associated with increased risk of insomnia and that PTSD is a significant contributor to overall morbidity in this population [27]. Further, our findings are consistent with prior research showing that burn survivors with PTSD report abnormal temperature sensitivity, and that the interplay between psychological and physical sequelae is a key determinant of long-term outcomes after burn injury [12,13,15,28]. Further, cognitive factors such as catastrophizing, negative appraisals, and maladaptive coping strategies mediate the relationship between PTSD and persistent pain or somatic complaints, reinforcing the role of neuropsychological pathways in symptom amplification [12,19,29].

PTSD-related hypervigilance and fear-avoidance behaviors may intensify symptom reporting and limit patients’ active engagement in rehabilitation therapies, perpetuating physical impairments like joint stiffness and reduced functional capacity. Additionally, PTSD-driven psychological distress might hinder therapeutic participation, leading to prolonged and intensified physical symptoms throughout the rehabilitation process [30].

The present study reinforces psychosomatic models suggesting that PTSD can amplify physical complaints through complex neuropsychological pathways. Multiple studies in burn populations demonstrate a bidirectional and entangled relationship between PTSD symptom clusters, particularly hyperarousal, intrusions, and emotional numbing, and increased pain interference and somatic symptoms, independent of pain intensity itself. These findings are consistent with psychosomatic models, which suggest that psychological distress can modulate physical symptom perception and chronicity via neurobiological mechanisms [12,13,19,28]. Recent work in psychoneuroimmunoendocrinology (PNIE) further elucidates that PTSD is associated with dysregulation across the central and autonomic nervous systems, immune and endocrine axes, and the gut–brain axis. PTSD is characterized by disruptions in the central and autonomic nervous systems, including altered stress reactivity, heart rate variability, and hypothalamic–pituitary–adrenal (HPA) axis function [31]. There is also consistent evidence of immune system dysregulation, with a pro-inflammatory state and elevated cytokines such as interleukin-6 and tumor necrosis factor-α, as well as altered neuroendocrine markers, including low basal cortisol and glucocorticoid receptor sensitivity [32,33,34,35,36]. Additionally, recent studies highlight the role of the gut–brain axis, with gut microbiota imbalances contributing to neuroinflammation and further modulating stress responses and somatic symptoms [31,32,37]. These interconnected pathways help explain the amplification and persistence of somatic symptoms in PTSD, especially in populations with significant physical trauma, such as burn survivors [31,32,38].

While our findings demonstrate a robust association between PTSD and increased physical symptom burden, the directionality of this relationship remains complex and likely bidirectional. On one hand, persistent somatic complaints following burn injuries may act as continuous reminders of the traumatic event, thereby contributing to the onset or maintenance of PTSD symptoms [13,19,20,39]. On the other hand, PTSD-related neurobiological alterations, including heightened stress reactivity, hypervigilance, and maladaptive coping mechanisms, may amplify the perception and persistence of physical symptoms [29,38,40]. This highlights the need for future longitudinal studies incorporating repeated assessments of psychological and physical symptoms over time, ideally beginning in the acute post-injury phase. Such designs would allow for a more precise delineation of temporal relationships and mediating factors. Additionally, the integration of biomarker-based assessments—capturing neuroendocrine, immunological, and autonomic dysregulation—may provide mechanistic insights into how PTSD and somatic symptoms interact and mutually reinforce each other in the context of burn recovery.

Clinically, our results emphasize the critical importance of early psychological assessments and systematic PTSD screening in burn rehabilitation settings. Integrated care models that concurrently address psychological and somatic symptoms could substantially improve patient outcomes. The routine tracking of physical symptoms in patients identified with PTSD would facilitate targeted interventions, potentially mitigating chronic symptom burden and enhancing overall rehabilitation efficacy, as early psychological distress is a strong predictor of long-term morbidity [41,42]. Future research should incorporate formal PTSD diagnoses using validated psychometric instruments to enhance diagnostic precision and comparability across studies. Intervention trials examining the efficacy of psychotherapeutic support in reducing physical symptom burdens during rehabilitation could further validate the integration of psychological care into burn treatment protocols [43].

Key strengths of this study include its multicenter, prospective design, rigorous standardization of symptom tracking, and clinically meaningful one-year follow-up period. However, several limitations should be considered. PTSD diagnosis was based on preexisting clinical diagnosis rather than standardized comprehensive assessment tools, potentially influencing diagnostic accuracy. Additionally, self-reported physical symptoms may introduce reporting bias. Also, an important limitation of our study is the fact that patients with PTSD had a significantly higher proportion of full-thickness burns and elevated ABSI scores, indicating relatively more severe injuries. This difference in injury severity may partially account for the increased burden of physical skin symptoms observed in the PTSD group, independent of psychological factors. Furthermore, the overall burn severity in our study cohort was moderate, with a mean TBSA of 15%, which may limit the generalizability of our findings to patients with more extensive burn injuries. In addition, the cohort’s demographic, predominantly male and characterized by occupational burn injuries, may limit generalizability. The limited number of female participants (n = 4) in our cohort precluded meaningful sex-stratified analyses, which may limit the ability to account for potential sex-related differences in symptom perception and reporting. Lastly, the absence of adjustment for confounding factors such as pre-existing chronic pain or depression necessitates the cautious interpretation of our findings.

## 5. Conclusions

This prospective multicenter cohort study demonstrates that patients with PTSD experience a significantly higher and sustained burden of physical skin symptoms following burn injuries compared to patients without PTSD. The persistent presence of symptoms such as xerosis, temperature sensitivity, numbness, and skin tightness at one-year post-rehabilitation underscores the clinical relevance of PTSD as a factor influencing long-term physical recovery outcomes. These findings highlight the importance of incorporating systematic PTSD screening and integrated psychotherapeutic interventions into burn rehabilitation protocols, aiming for comprehensive care that simultaneously addresses psychological trauma and somatic sequelae to optimize patient recovery and enhance quality of life.

## Figures and Tables

**Table 1:** Demographics and burn characteristics of all patients undergoing inpatient burn rehabilitation, dichotomized into patients with and without pre-existing PTSD diagnosis.

Characteristic	All Patients (n = 103)	Patients Without PTSD (n = 60)	Patients with PTSD (n = 43)	*p*-Value
**Demographics**				
Age, median, IQR (years)	44 [35–56]	42 [33–56]	48 [39–56]	0.39
BMI, mean ± SD (kg/m^2^)	27.13 ± 5.17	27.18 ± 4.74	27.06 ± 5.78	0.91
Sex (male/female)	99:4	60:0	39:4	**0.03**
**Etiology, N [%]**				
Flame	31 (30.4)	17 (28.3)	14 (32.6)	0.67
Scald	25 (24.5)	15 (25.0)	10 (23.3)	>0.99
Contact burn	15 (14.7)	6 (10.0)	9 (20.9)	0.16
Electrical	15 (14.7)	11 (18.3)	4 (9.3)	0.26
Explosion	6 (5.9)	4 (6.7)	2 (4.7)	>0.99
Friction burn	1 (1.0)	1 (1.7)	0 (0.0)	>0.99
Chemical	5 (4.9)	3 (5.0)	2 (4.7)	>0.99
Other/unknown	5 (4.9)	3 (5.0)	2 (4.7)	>0.99
**TBSA [%], median, IQR**				
Total	15 (6–25)	14 (7–25)	15 (6–25)	0.59
Superficial partial thickness	3 (0–10)	4 (0–10)	2 (0–8)	0.45
Deep partial thickness	7 (2–13)	6.5 (2–12)	8 (3–15)	0.31
Full thickness	0 (0–7)	0 (0–4)	2 (0–10)	**0.03**
**Other Characteristics**				
ABSI, median, IQR	5 (4–7)	5 (4–7)	6 (4–8)	**0.04**
Inhalation injury, N [%]	14 (13.6)	5 (8.3)	9 (20.9)	0.08
LOS acute care (days), median, IQR	26 (17–44)	23 (17–40)	29 (19–53)	0.16
LOS inpatient rehabilitation (weeks), median, IQR	4 (3–6)	3 (3–6)	5 (3–6)	0.09

Reported as n (%), unless stated otherwise. SD, standard deviation. IQR, interquartile range. TBSA, total body surface area. ABSI, Abbreviated Burn Severity Index. LOS, length of stay. Significance level *p* < 0.05 after significance correction using the Benjamini–Hochberg (BH) procedure. Statistically Significant *p*-values are highlighted in bold.

**Table 2:** Overview of physical symptom burden over time for patients with and without PTSD diagnosis at four assessment time points, ranging from T1 (beginning of rehabilitation), T2 (end of rehabilitation), T3 (3-month follow-up), to T4 (12-month follow-up). Displayed as absolute numbers, n (%). Statistically significant *p*-values are highlighted in bold.

Characteristic	All Patients (n = 103)	Patients Without PTSD (n = 60)	Patients with PTSD (n = 43)	*p*-Value
**Xerosis**				
T1	69 (67)	36 (60)	33 (77)	0.09
T2	68 (66)	46 (77)	22 (71)	0.61
T3	58 (60)	32 (55)	26 (68)	0.21
T4	55 (60)	27 (50)	28 (74)	**0.03**
**Sensitivity to Cold**				
T1	37 (36)	18 (30)	19 (44)	0.15
T2	41 (40)	24 (40)	17 (55)	0.19
T3	41 (43)	23 (40)	18 (47)	0.53
T4	42 (46)	19 (35)	23 (61)	**0.02**
**Sensitivity to Heat**				
T1	45 (44)	25 (42)	20 (47)	0.69
T2	46 (45)	28 (47)	18 (58)	0.38
T3	49 (51)	27 (47)	22 (58)	0.30
T4	45 (49)	21 (39)	24 (63)	**0.03**
**Skin Fragility**				
T1	65 (63)	38 (63)	27 (64)	>0.99
T2	66 (64)	45 (75)	21 (68)	0.47
T3	53 (55)	32 (55)	21 (55)	>0.99
T4	59 (64)	32 (59)	27 (71)	0.28
**Numbness**				
T1	41 (40)	22 (37)	19 (44)	0.54
T2	43 (42)	27 (45)	16 (52)	0.66
T3	41 (43)	19 (33)	22 (58)	**0.01**
T4	42 (46)	18 (33)	24 (63)	**0.006**
**Skin Tightness**				
T1	90 (87)	49 (82)	41 (95)	0.07
T2	84 (82)	48 (80)	25 (81)	>0.99
T3	73 (76)	40 (69)	33 (87)	**0.04**
T4	59 (64)	28 (52)	31 (82)	**0.004**
**Propensity to Sweat**				
T1	32 (31)	15 (25)	17 (40)	0.13
T2	35 (34)	21 (35)	14 (45)	0.37
T3	31 (32)	15 (26)	16 (42)	0.12
T4	27 (29)	10 (19)	17 (45)	**0.01**

Percentages are calculated based on the number of patients with available data at each time point. Due to loss to follow-up and incomplete data recording, the denominators vary over time as follows: All patients—T1: n = 103, T2: n = 103, T3: n = 96, T4: n = 92. Patients without PTSD—T1: n = 60, T2: n = 60, T3: n = 58, T4: n = 54. Patients with PTSD—T1: n = 43, T2: n = 31, T3: n = 38, T4: n = 38. Significance level *p* < 0.05 after significance correction using the Benjamini–Hochberg (BH) procedure. Statistically Significant *p*-values are highlighted in bold.

## Data Availability

The datasets generated and analyzed during the current study are available from the corresponding author upon reasonable request.

## Abbreviations

The following abbreviations are used in this manuscript:

PTSD	Post-Traumatic Stress Disorder
ICF	International Classification of Functioning, Disability, and Health
IES-R	Impact of Event Scale—Revised
BMI	Body Mass Index
TBSA	Total Body Surface Area
ABSI	Abbreviated Burn Severity Index
LOS	Length of Stay
IQR	Interquartile Range
SD	Standard Deviation
HPA	Hypothalamic–Pituitary–Adrenal (axis)
PNIE	Psychoneuroimmunoendocrinology
DGUV	Deutsche Gesetzliche Unfallversicherung
